# EpiSurf: metadata-driven search server for analyzing amino acid changes within epitopes of SARS-CoV-2 and other viral species

**DOI:** 10.1093/database/baab059

**Published:** 2021-09-29

**Authors:** Anna Bernasconi, Luca Cilibrasi, Ruba Al Khalaf, Tommaso Alfonsi, Stefano Ceri, Pietro Pinoli, Arif Canakoglu

**Affiliations:** Dipartimento di Elettronica, Informazione e Bioingegneria, Politecnico di Milano, Via Ponzio 34/5, Milano 20133, Italy; Dipartimento di Elettronica, Informazione e Bioingegneria, Politecnico di Milano, Via Ponzio 34/5, Milano 20133, Italy; Dipartimento di Elettronica, Informazione e Bioingegneria, Politecnico di Milano, Via Ponzio 34/5, Milano 20133, Italy; Dipartimento di Elettronica, Informazione e Bioingegneria, Politecnico di Milano, Via Ponzio 34/5, Milano 20133, Italy; Dipartimento di Elettronica, Informazione e Bioingegneria, Politecnico di Milano, Via Ponzio 34/5, Milano 20133, Italy; Dipartimento di Elettronica, Informazione e Bioingegneria, Politecnico di Milano, Via Ponzio 34/5, Milano 20133, Italy; Dipartimento di Elettronica, Informazione e Bioingegneria, Politecnico di Milano, Via Ponzio 34/5, Milano 20133, Italy

## Abstract

EpiSurf is a Web application for selecting viral populations of interest and then analyzing how their amino acid changes are distributed along epitopes. Viral sequences are searched within ViruSurf, which stores curated metadata and amino acid changes imported from the most widely used deposition sources for viral databases (GenBank, COVID-19 Genomics UK (COG-UK) and Global initiative on sharing all influenza data (GISAID)). Epitopes are searched within the open source Immune Epitope Database or directly proposed by users by indicating their start and stop positions in the context of a given viral protein. Amino acid changes of selected populations are joined with epitopes of interest; a result table summarizes, for each epitope, statistics about the overlapping amino acid changes and about the sequences carrying such alterations. The results may also be inspected by the VirusViz Web application; epitope regions are highlighted within the given viral protein, and changes can be comparatively inspected. For sequences mutated within the epitope, we also offer a complete view of the distribution of amino acid changes, optionally grouped by the location, collection date or lineage. Thanks to these functionalities, EpiSurf supports the user-friendly testing of epitope conservancy within selected populations of interest, which can be of utmost relevance for designing vaccines, drugs or serological assays. EpiSurf is available at two endpoints.

**Database URL**: http://gmql.eu/episurf/ (for searching GenBank and COG-UK sequences) and http://gmql.eu/episurf_gisaid/ (for GISAID sequences).

## Introduction

With the coronavirus disease 2019 (COVID-19) pandemic outbreak, unprecedented efforts have been dedicated to the sampling and sequencing of the severe acute respiratory syndrome coronavirus 2 (SARS-CoV-2), with the objective of capturing and then studying SARS-CoV-2 variations and their effects. Leveraging on our previous experience on human genomics-targeted computational systems (1), we have directed our interest towards the integration, curation, search and analysis of viral sequences, yielding several contributions: a conceptual model for describing viral sequences with their metadata and variants (2), an integrated search system for viral sequences (3), a data visualization application (4) and a knowledge base for studying variant effects (5). Capitalizing on the above experiences and resources, we developed and hereby present a Web-based search application for studying epitopes in the context of viral sequences that have been so far deposited worldwide.

Epitopes are strings of amino acid residues from a pathogen’s protein that can be recognized by antibodies or B/T cell receptors, thus activating an immune response from the host; in particular, epitopes available for the Spike protein of SARS-CoV-2 are used in the design of COVID-19 vaccines. For epitope-based vaccine design, it is important to study their conservation; conversely, observing epitope variability has applications in disease monitoring, diagnostic settings and drug design. We adopt the most basic conservancy measure for an epitope (i.e. a region on a protein), based on the number of changed amino acid residues with respect to the reference sequence of the virus species in the same position range. ‘Conserved’ epitopes have a zero distance from the reference, whereas ‘modified’ epitopes exhibit at least one amino acid change.

To date, the most relevant resource for epitopes employed by the research community is the Immune Epitope Database (IEDB) (6). It encompasses immune epitope data of a large number of species, including antibody, T cell and major histocompatibility complex (MHC) binding contexts associated with several diseases. EpiSurf imports all epitopes available from IEDB; in addition, EpiSurf supports user-defined epitopes, intended as position ranges on specific virus proteins.

EpiSurf supports the search of viral sequences deposited on public platforms—released day by day on GenBank, COG-UK and GISAID and then integrated within the ViruSurf platform; relevant sequences can be extracted, thanks to a rich set of metadata information, including the sampling location, collection and deposition date, sequence’s lineages and strains, and submission laboratory. On top of this, EpiSurf provides several methods to intersect selected sequences and selected epitopes, thereby integrating information about amino acid changes and epitopes extracted from the largest and most popular data collections in the world using their metadata. Lastly, the integration with the VirusViz tool allows informative visualization of sequence variation in the specific epitopes’ locations. Building up on our previously developed resources, EpiSurf offers a novel, fully independent, integrated environment for evaluating conservancy of epitopes against arbitrarily extracted viral populations, reflecting the spreading of viruses in time and space and their genetic evolution.

## Comparison with existing systems

Several tools for epitope prediction have been studied in the past (7). The most used and well-known resource in this field is the suite of IEDB (6), comprising a set of T Cell and B Cell Epitope Prediction tools (see http://tools.iedb.org/main/).

For the specific case of SARS-CoV-2 (and closely related viruses), we report the following. COVIEdb (8) targets pancoronavirus vaccine development, by describing a database of potential B/T cell epitopes for SARS-CoV-2, SARS-CoV, Middle East respiratory syndrome (MERS)-CoV and RaTG13-CoV; database entries are predicted by using tools hosted by IEDB, exploiting the similarity of other viruses [as proposed by Grifoni *et al.* (9)]. Similar databases are provided in CoronaVIR (10), a database of coronavirus virulent glycoproteins (DBCOVP) (11) and CoronaVR (12). COVID miner (13) and COVID profiler (14) provide companion vaccine design tools, with a focus on prediction (the latter also providing light integration with IEDB data).

EpiSurf is not intended for epitope prediction. Instead, it may be labeled as a tool for conservancy and population coverage analysis. IEDB curates a collection of tools (http://tools.iedb.org/main/analysis-tools/), used for a variety of detailed analyses. Among these, EpiSurf is similar in spirit to the Epitope Conservancy Analysis (ECA) tool (15); however, there the user must provide the amino acid sequences of (i) all the epitopes to be tested and (ii) all the virus sequences whose changes should be tracked, whereas EpiSurf offers a seamless integration with all public sequences and variants currently available from GenBank, COG-UK and GISAID.

COVIDep (16) is an integrative effort more similar in the approach to EpiSurf, as it joins IEDB epitopes with regularly updated GISAID sequences. The proposed ‘Population coverage analysis’ is an interesting view providing quantifications of ‘conservation’ and ‘population coverage’ for each epitope. However, the provided epitopes are those that were predicted and experimentally derived (based on SARS-CoV data) at the time of publication (May 2020) by the authors of the work. This important exercise resulted into a total of 284 T cell epitopes and 58 B cell linear epitopes. On the contrary, EpiSurf keeps its list of epitopes updated, now reaching 3690 T cell epitopes, 1006 MHC Ligand epitopes and 1421 B cell epitopes—see Table 2. Comparatively, EpiSurf offers a scalable approach to epitope conservancy analysis that is very useful as we expect that new sequences and epitopes will be deposited for a long time. Moreover, the COVIDep resource provides much less freedom of choosing metadata for sequences and epitopes, no possibility of fixing specific amino acid changes, and no ways of analyzing in detail the sequences that mutate on the epitope. EpiSurf, on the contrary, does offer all sequence metadata in its result table and complements it with a table for understanding the breakdown of statistics over the different metadata of the population (for both EpiSurf and EpiSurf-GISAID) as well as VirusViz visualization functionalities (for EpiSurf).

The Virus Pathogen Database and Analysis Resource (ViPR) (17) is another important tool that connects (both predicted and experimentally derived) epitopes and proteins of sequences deposited in the GenBank, whereas no link to GISAID is provided. EpiSurf is novel in that it offers several aggregations and simple statistics on both GenBank and GISAID data.

The COG-UK Mutation Explorer (COG-UK-ME) (18) recently released an interface dedicated to only UK data and its variation (also in the context of T cell epitopes reported by experimental studies).

Table 1 summarizes the relevant aspects of the tools that allow population conservancy and coverage analysis. Recently, they focused on SARS-CoV-2 and human hosts; however, ViPR and IEDB ECA pre-existed, offering support to many kinds of viruses. Only ViPR offers the possibility to select sub-populations of sequences at the user’s preference. COG-UK-ME focuses on T cell epitopes. Sources for epitopes are various: IEDB ECA only allows user input strings, whereas ViPR and EpiSurf enrich them with the IEDB corpus of epitopes. COVIDep, ViPR and COG-UK-ME currently offer curated lists, respectively, predicted from SARS-CoV, predicted with NetCTL (19) and manually extracted from experimental studies.

**Table 1.** Comparison of resources for analyzing epitopes over a sequence population—for each system, we indicate the data source of SARS-CoV-2 sequences; the presence of sequences from other viruses; which hosts are considered; if sub-populations of sequences can be selected based on metadata; which epitope assays are considered; the data source for their extraction; and the presence of aggregation/visualization methods for showing mutations in the context of epitopes

	**SARS-CoV-2 seq.**	**Other viruses**	**Hosts**	**Seq. filters**	**Epitope assay type**	**Epitope source**	**Vis/Agg. on epit.**
IEDB ECA (15)	User input	Virus agnostic	Host agnostic	–	B cell; T cell; MHC	User input	–
COVIDep (16)	GISAID	–	Human	Location	B cell (linear); T cell	Pred. from SARS-CoV	–
ViPR (17)	GenBank	All in GenBank	All in GenBank	Full metadata	B cell; T cell; MHC	User input; IEDB; pred. (NetCTL)	–
COG-UK-ME (18)	COG-UK	–	Human	–	T cell	Collected from exp. studies	With ggseqlogo
EpiSurf	GenBank; COG-UK	SARS; MERS;	All in GenBank	Full metadata	B cell; T cell; MHC	User input; IEDB	With VirusViz
	GISAID	dengue; Ebola					

For data visualization, EpiSurf provides a connection to VirusViz (4) (http://gmql.eu/virusviz/), a Web application for visualizing and exploring the fully open source nucleotide and amino acid changes that are made available through search services or autonomously provided as input from users. When VirusViz is opened starting from an EpiSurf search, the tool visualizes a bar plot where the *x*-axis represents the amino acid positions of a protein, bars’ heights represent the number of sequences in the selected populations that feature a change in the bar’s position and epitope position ranges are represented as blue vertical regions (see Figure 6 in Example 3).

## Materials and methods

### Database schema

**Figure 1.**
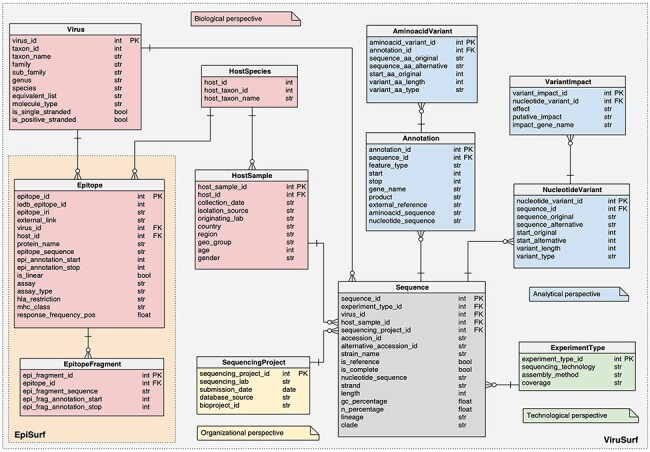
Logical schema of the relational database in the back-end of EpiSurf.

The database schema, represented in Figure 1, is partially inherited from ViruSurf (3) (http://gmql.eu/virusurf/), an integrated database of SARS-CoV-2 sequences (and of other similar viruses), storing all the sequences deposited to GenBank (20) and COG-UK (21). A dual database (http://gmql.eu/virusurf_gisaid/) stores relevant metadata and variation information of GISAID sequences (22).

For both databases, the schemas are centered on the SEQUENCE, described by biological metadata (VIRUS and HOSTSAMPLE), technological metadata (EXPERIMENTTYPE) and organizational metadata (SEQUENCINGPROJECT). The ‘analytical perspective’ provides the ANNOTATION, AMINOACIDVARIANT, NUCLEOTIDEVARIANT and VARIANTIMPACT tables.

EpiSurf and EpiSurf-GISAID feature two novel databases, whose complete schema descriptions are available at https://github.com/DEIB-GECO/EpiSurf/wiki/Database-and-sources, pointing to SchemaSpy (http://schemaspy.org/) documents. In the following, we only detail the additions that were not present in (3):

1. The HOSTSPECIES table, a connector between the HOSTSAMPLE and the EPITOPE tables, representing the identification of the animal species both involved in the extraction of biological material to be sequenced and in the epitope design.
2. The EPITOPE table, describing the epitopes extracted from IEDB, connected both to HOSTSPECIES and to the VIRUS table. The core information is contained within the ‘epitope sequence’*—*the amino acid sequence of the epitope, starting at position ‘epi_annotation_start’ and finishing at position ‘epi_annotation_stop’ of the reference sequence of a given protein, referred as ‘protein_name’; the ‘is_linear’ attribute defines continuous (true) or discontinuous (false) epitopes, composed of amino acid residues that may be located in different protein regions—brought together by protein folding. We also report information on the experiments performed to retrieve the epitope. Each epitope record in EpiSurf may correspond to multiple records from IEDB (each assigned to one single experiment, i.e. assay). Therefore, the following four fields can take multiple values:
  - ‘assay’ indicates the target of the experiment [allowing values (‘T cell’, ‘B cell’, ‘MHC ligand’)];
  - ‘assay_type’ indicates the outcome—considering possibly multiple experiments (we have ‘positive’, ‘negative’ and ‘both’, when positive and negative outcomes were included);
  - ‘hla_restriction’ (also referred to as ‘mhc allele’) indicates the list of the class (e.g. ‘HLA Class I’) or lists of alleles (e.g. ‘HLA-B*35:01, HLA-B*15:01’) to which the epitope is restricted—this is relevant only for T cell and MHC ligand assays;
  - ‘mhc_class’ indicates the general classes of alleles provided in the previous field (possible values are ‘I’, ‘II’ or ‘I,II’ if both Class I and II alleles are considered).
3. Finally, we add the ‘response_frequency_pos’: on IEDB this measure is defined as the number of positively responded subjects (*R*) divided by the total number of those tested (*N*), summed up by mapped epitopes; however, to compensate for epitopes that are identified by a low number of assays, we employ a corrected formula [proposed in (23)] resulting as (*R*−√*R*)/*N*, where the importance of corrections decreases as the number of assays increases.
4. The EPITOPEFRAGMENT table, which contains the segments (identified by the ‘epi_fragment_id’) of nonlinear epitopes (each of which is contained within one comprehensive epitope). In the case of linear epitopes, we store a unique fragment in this table. The ‘epi_fragment_sequence’ contains the amino acid sequence of the single fragment starting at ‘epi_frag_annotation_start’ and finishing at ‘epi_frag_annotation_stop’.

### Database content

#### Content imported from ViruSurf

EpiSurf database is fueled by the same automatic import pipeline that frequently extracts and processes sequences, metadata and variant information for populating the ViruSurf database (3). Specifically, we extract sequences and their metadata from COG-UK and GenBank, whereas we compute amino acid changes according to the following steps: (i) for each virus species, selection of a reference sequence and a set of annotations; (ii) for each sequence, computation of the optimal global alignment to the reference by means of the Needleman–Wunsch (NW) algorithm (24); (iii) identification of the sub-sequences corresponding to the reference annotations; (iv) translation of coding regions into their equivalent amino acid sequences; (v) alignment of translated amino acid sequences to the corresponding reference amino acid sequences (also using NW) and (vi) inference of amino acid changes. Thanks to a Data Connectivity Agreement with GISAID, we have access to frequently updated information downloaded from the EpiCoV^TM^ database including, for each sequence, selected metadata and all amino acid changes.

#### Content imported from IEDB

We regularly download and process experimental epitope sequences and their metadata. The process is controlled by an automated pipeline that retrieves the DB exports of the B cell, T cell, and MHC ligand in the form of CSV files from the IEDB Database Export site (https://www.iedb.org/database_export_v3.php) at the section ‘CSV Metric Export’. After extraction, each file is parsed as regular tabular data, which allows for the easy selection of the relevant characteristics. Indeed, the attributes available in our database are copied ‘as is’ from the origin, with the exception of attributes regarding assays. As mentioned in the discussion of the EPITOPE table, the four attributes ‘assay’, ‘assay_type’, ‘hla_restriction’ and ‘mhc_class’*—*concerning a single assay on IEDB—are concatenated in a single epitope in EpiSurf. Similarly, the ‘response_frequency_pos’ is calculated as an aggregation over all the positive assays that derived the epitope.

The pipeline associates three foreign keys to each imported epitope: the ‘virus_id’, the ‘protein_name’ and the ‘host_id’. The first two attributes are derived by directly mapping the virus name and the UniProtID, respectively, to the ‘id’ of the organism in the VIRUS table and to the product in the ANNOTATION table. The third attribute links an epitope to a host in the table HOSTSPECIES. To make sure that this foreign key can be set, before the import stage, we automatically update the hosts’ table by collecting from the NCBI Taxonomy database the ‘name’ and ‘id’ of the species that are not already available in ViruSurf. Finally, we generate one row inside the EPITOPEFRAGMENT table for every epitope sequence, be it linear or non-linear, and link them through the key ‘epitope_id’ to the EPITOPE table. In this way, it is easy to access all epitope sequences by a regular join of the two tables and selecting the ‘epi_fragment_sequence’.

#### Quantitative description

Table 2 provides a description of the current EpiSurf content; for each virus we report the rank, the NCBI Taxonomy identifier/name and the number of sequences included from each source. In the last four columns, we provide the number of epitopes retrieved from IEDB for the indicated species. The total number is broken down into three categories: T cell, B cell and MHC ligand epitopes. The most substantial contribution in the database is provided by SARS-CoV-2 data; however, the system works seamlessly also for SARS-CoV, MERS-CoV, Ebola and dengue species. In the future, additional viruses may be added with small changes in the configuration of pipelines and no changes in the data representation and query engine.

**Table 2.** Summary of EpiSurf content as of 18 July 2021. For each taxon name (identified by a taxon ID and rank) and each source, we specify the number of distinct sequences and the number of available epitopes, with their breakdown into T cell, B cell and MHC ligand assays

					**IEDB epitopes**			
**Taxon rank**	**Taxon ID**	**Taxon name**	**Source**	**#Seq.**	**#Total**	**#T cell**	**#B cell**	**#MHC lig.**
No rank	2 697 049	Severe acute respiratory syndrome coronavirus 2	GISAID	2 390 870				
No rank	2 697 049	Severe acute respiratory syndrome coronavirus 2	GenBank	691 734	6117	3690	1421	1006
No rank	2 697 049	Severe acute respiratory syndrome coronavirus 2	COG-UK	574 061				
Species	694 009	Severe acute respiratory syndrome-related coronavirus	GenBank	674	1722	782	437	503
Species	1 335 626	Middle East respiratory syndrome-related coronavirus	GenBank	1453	110	110	–	–
Species	2 010 960	Bombali ebolavirus	GenBank	8	–	–	–	–
Species	565 995	Bundibugyo ebolavirus	GenBank	22	14	–	14	–
Species	186 539	Reston ebolavirus	GenBank	58	–	–	–	–
Species	186 540	Sudan ebolavirus	GenBank	39	536	240	9	287
Species	186 541	Tai Forest ebolavirus	GenBank	9	–	–	–	–
Species	186 538	Zaire ebolavirus	GenBank	2932	2113	700	487	926
Strain	11 053	Dengue Virus 1	GenBank	12 059	1631	1130	215	286
Strain	11 060	Dengue Virus 2	GenBank	9646	2024	1396	322	306
Strain	11 069	Dengue Virus 3	GenBank	5628	2318	1704	224	390
Strain	11 070	Dengue Virus 4	GenBank	2812	1090	782	96	212

IEDB epitopes.

### Data access optimization

Several optimization steps were designed for allowing acceptable query performances. As the most critical part of the system involves data on SARS-CoV-2 virus and a human host, the data on epitopes and matching variants regarding this fraction of the database have been precalculated into several materialized views, one for each of the 12 distinct proteins in the system (i.e. ORF1a, ORF1ab, Spike, ORF3a, E, M, ORF6, ORF7a, ORF7b, ORF8, N and ORF10) and 16 sub-proteins (from NSP1 to NSP16).

### System development and sustainability

In terms of the software architecture, EpiSurf is organized as a Web application where the back-end runs on a Flask (Python) server, and the front-end is implemented with the Javascript Vue.js framework. The underlying relational database is built with PostgreSQL (Version 10.17); continuous interactions with the database are handled with the Python sqlalchemy library. The code is available on GitHub at https://github.com/DEIB-GECO/EpiSurf/.

The objective of EpiSurf is to offer a concrete public endpoint for research on the interplay of epitopes with current viral sequences; its sustainability depends on the timely provision of both sequences and epitope inputs, as well as on the interplay with the ViruSurf database and VirusViz visualization application. Sequence data are currently updated on EpiSurf and EpiSurf-GISAID weekly; SARS-CoV-2 epitopes from IEDB are updated with the same frequency. We also periodically consider other species (SARS-CoV, MERS, dengue and Ebola).

## Results

**Figure 2.**
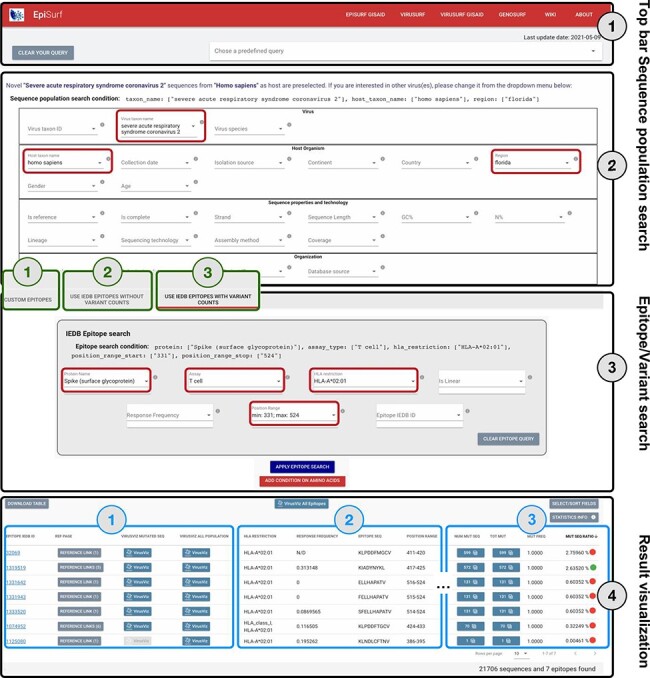
Overview of the EpiSurf interface, divided into four parts (see black rectangles). ‘Part 1’ (Top bar) allows clearing all filters selected in the interface or selecting predefined example queries. ‘Part 2’ (Sequence population search) is used for selecting sequences of interest. SARS-CoV-2 and human host are pre-selected, whereas the ‘Florida’ location has been added as an example (see red rectangles). After metadata search, users select between three different modes (squared in green): (1) Custom epitopes (shown in detail in Figure 3); (2) Use IEDB epitopes without variant counts; (3) Use IEDB epitopes with variant counts. For Parts 3 and 4 of this figure, we assume the selection of Modes 2 or 3, whereas Figure 3 shows Parts 3 and 4 after selecting Mode 1. ‘Part 3’ (Epitope/Variant search) enables selecting epitopes from IEDB. The Spike protein is pre-selected (but users can easily change the choice of protein), whereas other conditions allow filtering the epitopes by using metadata available in IEDB. As an example, we show the selection of T cell assay, HLA-A*02:01 restriction and a position range covering the receptor binding domain (25)—see red rectangles. Finally, ‘Part 4’ (Result visualization) provides a table describing selected epitopes, further vertically decomposed into three areas (shown in blue): ‘Area 1’ includes a number of buttons to open the results within the IEDB page, VirusViz (considering only sequences mutated on the epitope range or all the ones in the population selected in Part 2); ‘Area 2’ includes sortable and adjustable metadata about epitopes; and ‘Area 3’ is present only in Mode 3 and includes counters that define the conservancy of the epitope in the population of interest. The columns of the table are customizable and the full table can be conveniently downloaded as a CSV file. The two most relevant counts in the search, i.e. the number of sequences and of epitopes, are provided at the bottom right corner of the web page.

The web interface of EpiSurf is composed of four sections, numbered in Figure 2 (i) the menu bar, for accessing services, documentation and predefined example queries; (ii) the search interface over sequence metadata attributes; (iii) the search interface over epitopes, available in three modes (user-defined epitopes, IEDB epitopes with—and without—the calculation of statistics on variants) and (iv) the results section, showing epitopes with their metadata, counters and visualization options. Users should select exactly one host organism and one virus (pre-selected options are ‘homo sapiens’ and ‘SARSCoV-2’), as this is the default configuration for matching sequences with epitopes. Interaction over (2) and (3) is carefully designed in the three modes, as the underlying system builds complex queries that intersect the sequences resulting from (2) with the epitopes resulting from (3) by considering the amino acid changes exhibited by sequences within given epitope ranges.

### Sequence population search

The Metadata search section is organized in four parts: ‘Virus’ and ‘Host Organism’ (from the ‘biological’ perspective of the database schema), and ‘Technology’ and ‘Organization’ (from the corresponding perspectives). It includes attributes that are present in most of the sources, described by an information tab that is opened by clicking on grey circles; values can be selected using drop-down menus. At the side of each value, we report the number of sequences in the repository with that value. The user can select the desired sequence population by entering values from all the drop-down menus; the result is the set of sequences matching all the filters. For numerical fields (age, length, GC% and *N*%), the user must specify a range between a minimum and maximum value; in addition, the user can check the Not Defined (N/D) flag, thereby including in the result those sequences having an unknown value. Similarly, ranges of collection and submission dates can be selected using calendar-like drop-down components, also supporting the N/D flag.

### Epitope search

Interaction over epitope data can be conducted using three different modes, respectively, for inputting user-defined epitopes, for extracting epitopes as they are imported from IEDB and for associating to those epitope statistics computed over the mutated sequences of the database. We detail the three scenarios in the following.

#### Mode 1: custom epitopes

**Figure 3.**
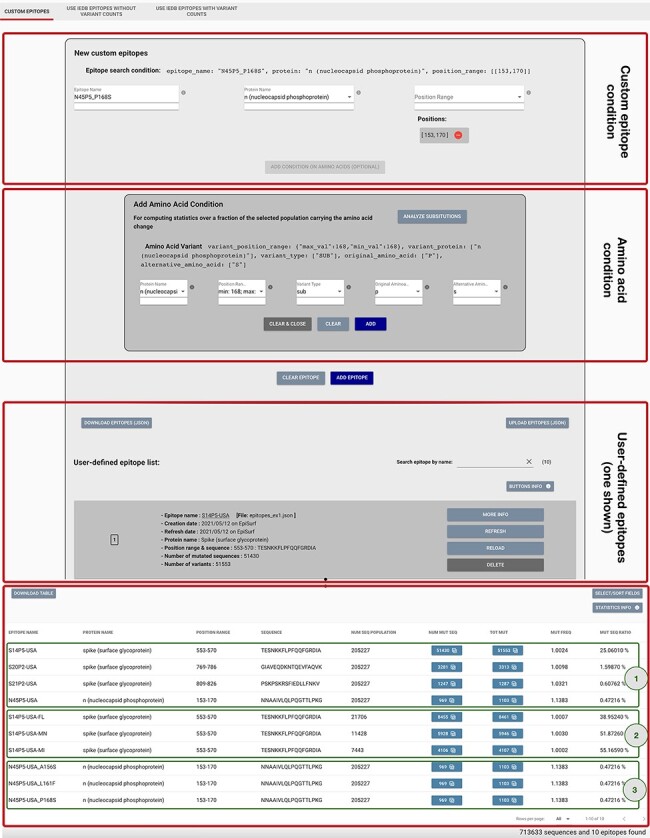
Epitope definition panels in the ‘Custom Epitope’ Mode 1, where users can input their defined epitopes, inserting a name, the protein and range (or collection of ranges, when the proposed epitope is nonlinear). In addition, the user can optionally add an amino acid condition for restricting the population selected in the Sequence Population Search panel (see Figure 2) to the viruses carrying a specific amino acid change. Once created, epitopes are added to a list, displayed on the page; each epitope information can be inspected by using the MORE INFO button, updated with the REFRESH button, modified with the RELOAD button or removed with the DELETE button. Epitopes can be downloaded and then uploaded during a different session of the EpiSurf use. The bottom table shows the results of the epitope design session, as further described in Example 1 of the Use Cases section.

This mode is particularly useful in the context of B cell epitopes, as these can be examined regardless of the human leukocyte antigen (HLA) restriction on the targeted population. The user is provided with a panel, shown in Figure 3, for defining candidate epitopes by providing its name, a specific protein on the virus and a position range (possibly discontinuous) on the protein. The epitope may be added to the list as is; in this case, the statistics will be computed over the full sequence population selected with the Metadata search.

Optionally, the user may select an additional condition on one amino acid change, with the purpose of instructing the system to compute statistics over the fraction of the selected population that carries the amino acid change. The panel allows the selection of a specific protein, a range of coordinates, a type of variation (insertion, deletion or substitution), an original amino acid residue and the corresponding alternative residue. The filter selection may be approved (ADD), deleted for choosing alternative ones (CLEAR) or deleted for removing the entire amino-acid-related condition (CLEAR and CLOSE).

The choice of amino acid filters is supported by a practical add-on triggered by the ‘Analyze Substitutions’ button, which allows the inspection of the characteristics of a specific replacement from the original into an alternative amino acid. Each involved (source or target) residue is characterized by a series of structural categorical properties (such as polarity, charge and flexibility) and of numerical properties (e.g. molecular weight and hydrophobicity); the pair of residues involved in the change is associated to a measure of its impact (Grantham distance (26)); a threshold on impact maps a change into radical or conservative categories.

After addition, the new epitope is inserted in a list of user-defined epitopes, which are presented by providing summary information, including its name, creation and refresh date, protein and position range, and virus/host taxon name and number of mutated sequences and of variants. Current epitopes in the list can be downloaded as a JSON file, thereby supporting the possibility of reloading specific files representing the status of saved interaction with EpiSurf; in this way, users may organize and manage the information collected about user-defined epitopes through many sessions of EpiSurf use.

Out of the current epitope list, users may read more information on the epitope (MORE INFO); refresh its counters (REFRESH)—this option is typically used after uploading an external JSON file as discussed above; reload all the values (originally used to create that epitope) into the drop-down menus of the sequence and epitope search panels (RELOAD)—this option facilitates the creation of a new epitope with different coordinates or for testing its conservancy on a different underlying population; and finally delete the element from the list (DELETE).

The result table stores all the relevant information on the defined epitopes connecting them with statistics on the sequences mutated on each epitope’s range. The table can be downloaded for subsequent data analysis as a CSV file. The last five columns of the table describe (i) ‘NUM SEQ POPULATION’: the number of sequences available in the population where the epitope has been tested in EpiSurf (i.e. matching the filters in the Metadata and Amino Acid Condition columns); (ii) ‘NUM MUT SEQ’: the number of sequences in the selected population that has at least one amino acid change exactly matching with the epitope position range; (iii) ‘TOT MUT’: the number of total amino acid changes exhibited by the full population of sequences (note that any insertion counts for one); (iv) ‘MUTATED FREQ’: the ratio of total variants (iii) over the number of mutated sequences (ii) and (v) ‘MUTATED SEQ RATIO’: the ratio of mutated sequences (ii) over the total of the selected population (i). When epitopes have been defined also using an amino acid condition, Counters (ii) and (iii) are computed by considering the fraction of the population that exhibits the specific selected amino acid condition.

By clicking on the ‘NUM MUT SEQ’ number, the list of mutated sequences with their metadata is shown in a table. From here, EpiSurf users may invoke VirusViz that will be opened on a variant distribution that considers all the mutated sequences and highlights the chosen epitope. By clicking on the ‘TOT MUT’ number, the user will open a new panel called ‘Epitope mutation statistics’, where the number of mutated sequences can be observed in a custom breakdown, grouping by several attributes concerning location, collection time and phylogenetic classification methods. A table is generated providing, for each specific amino acid change in a row, the number of sequences exhibiting such a change in each formed group.

#### Mode 2: IEDB epitopes without variant count

Modes 2 and 3 take advantage of the epitopes publicly deposited to IEDB. In both modes, the user can select epitopes by using seven different drop-down menus, representing metadata attributes of epitopes, extracted from IEDB: the protein, the type of experiment performed to recognize the epitope, the presence of specific HLA restrictions, if it is linear or discontinuous, the allowed range of corrected response frequency, a range of coordinates (i.e. all epitopes overlapping with the coordinate range are selected) and the specific epitope identifier within IEDB. The selection condition is determined as a conjunction of the filters selected in each drop-down menu; the protein and position range accept a single value as a filter and the other attributes accommodate multiple values (intended in disjunction).

The results in the bottom panel are in a tabular format; each row of the table represents one epitope, with links that refer it back to IEDB pages and relevant metadata (columns can be sorted and selected/deselected); the full table can be downloaded as a CSV file. VirusViz may be invoked on (i) the full population of sequences and all epitopes in the results (using the button at the top of the table) and (ii) the full population of sequences and one specific epitope (using the button on the epitope’s row). In both cases, users should invoke the visualizer after selecting populations of small size.

#### Mode 3: IEDB epitopes with variant count

This mode adds to Mode 2 the computation of statistics. It is the most sophisticated use of EpiSurf and requires a heavier computational load on the back-end (therefore, in addition to selecting small populations, users are also suggested to select a small number of epitopes). The specific feature introduced by this mode is the addition of an amino acid filter, which includes a variant position, type, original and alternative amino acid residue; its effect is to restrict the calculation of the four statistics only to sequences that exhibit matching amino acid changes. Changes may be chosen only among positions that are allowed by the previously set position range filter. As in Mode 1, we provide the ‘Analyze Substitutions’ functionality to aid users in evaluating the characteristics of amino acid replacements.

As in Mode 2, the result table provides links to IEDB or to invoke VirusViz and entries describing metadata from IEDB; in addition, as in Mode 1, the result table provides in the last four entries a quantitative description of sequence changes over each epitope: (i) ‘NUM MUT SEQ’: the number of sequences in the selected population that exhibits at least one amino acid change within the epitope position range; (ii) ‘TOT MUT’: the number of total amino acid changes exhibited by the full population of sequences; (iii) ‘MUT FREQ’: the ratio of total variants (ii) over the number of mutated sequences (i) and (iv) ‘MUT SEQ RATIO’: the ratio of mutated sequences (i) over the total of the selected population.

For this mode, we have designed a mechanism that ensures that users carefully select epitopes to be intersected with the population of interest. Indeed, while for B cell epitopes no attention is needed w.r.t. alleles expressed in the population, much concern should be dedicated when T cell or MHC ligand epitopes are targeted. In these cases, we recommend considering epitopes with a high response frequency, by setting a threshold—whose value was suggested by experts to be at least 0.2—using the response frequency provided by IEDB. We opted to use this threshold with the corrected formula proposed in (23), being the threshold even more conservative in this case. Besides this, users should consider the percentages of ‘MUT SEQ RATIO’ with care, ensuring that the HLA restriction is appropriate for the observed population [by checking suitable population allele databases, e.g. the Allele Frequency Net Database (27)]. Note that we have assigned a color code to support users in understanding how much they can rely on the observed statistics:

- ‘green’ denotes epitopes that have been derived by B cell assays and/or by T cell/MHC ligand assays with a positive assay response frequency ≥ 0.2;
- ‘orange’ denotes epitopes that have been derived by reliable assays (i.e. B cell assays or T cell/MHC ligand assays with response frequency ≥ 0.2) but also by less reliable assays (i.e. T cell/MHC ligand assays with a response frequency *< *0.2);
- ‘red’ denotes epitopes that have been derived by T cell/MHC ligand assays with a positive assay response frequency *< *0.2.

Similar to Mode 1, the user may click on ‘NUM MUT SEQ’ and ‘TOT MUT’ numbers to activate further analysis features.

### GISAID-specific EpiSurf

EpiSurf presents a version that is specific for the data imported from GISAID, as the data agreement does not allow merging GISAID information with information from other sources. The GISAID version has a panel for population selection offering restricted options, otherwise Modes 1, 2, and 3 are available with no change, except that VirusViz buttons are not available. Of course, since amino acid variants are sourced from different databases, epitope mapping to amino acid variants produces different counts in the two systems.

## Use cases

### Example 1

 Amrun *et al.* (28) present four different immunodominant B cell assay epitopes, to be used as highly specific and sensitive serological diagnostic targets, i.e. to test for the presence of the virus in patients potentially exposed to SARS-CoV-2. The candidate epitopes are named S14P5, S20P2 and S21P2 on the Spike protein, and N4P5 on the N protein. In their study (28), authors studied the conservation of these epitopes across 17 000 SARS-CoV-2 sequences publicly available at the time of writing. They reported low rate of potential amino acid changes over the epitopes.

By using Mode 1 of EpiSurf (Custom Epitopes), it is possible to replicate such epitopes as ranges of positions on the proteins, respectively, on [553–570], [769–786] and [809–826] on Spike and [153–170] on N. These may be checked, for instance, against the EpiSurf sequence population from the USA, of about 205 000 sequences as of 9 May 2021. Figure 3 shows a particular snapshot of the analysis session where the user has already inserted all four epitopes. See the third red rectangle framing the user-defined epitopes, where—for brevity—we only show the card produced for the first S14P5 epitope. When all four have been inserted, the user will be provided with the results framed by the green rectangle (1). It is worth noting that the first epitope has a high ratio of altered sequences, i.e. 25.1%. The user may be interested in inspecting the breakdown of such a consistent set of sequences. By clicking on the number of total amino acid changes (i.e. ‘TOT MUT’), we open the ‘Epitope mutation statistics’ functionality. We may group by the country attribute, thereby obtaining a table that, for each amino acid change, reports the total of sequences exhibiting such changes, and the breakdown of such an amount by ‘country’. Through sorting by descending total count, we observe that the Spike A570D position is the most commonly mutated one. We also check the most impacted US states (grouping by the attribute ‘region’), which are Florida, Minnesota and Michigan. An alternative grouping can be performed on ‘lineage’, highlighting that the sequences with this mutation are almost always assigned to the B.1.1.7 lineage, corresponding to the Variant of Concern [first defined on a Virological.org post in December 2020 (29)]. We can make our conservancy analysis more specific by adding new epitopes to our list, tested against smaller populations; in Figure 3, Result section, box (2), we have created the candidate epitopes S14P5-USA-FL, S14P5-USA-MN and S14P5-USAMI. In the ‘MUT SEQ RATIO’ column, we observe that Minnesota (MN) and Michigan (MI) have a higher incidence of mutations on this epitope.

We then focus on N4P5 [reported in (28) as the most stable epitope out of the four]. In our knowledge base [a corpus of variants’ annotations regarding their increased/decreased effects on kinetics/epidemiology/immunology levels, obtained through a systematic search of the published or preprint literature (5)], we find three amino acid changes falling within the scope of this epitope (namely, A156S, L161F and P168S); it has been claimed that they may lower the protein stability and modify the protein flexibility (30). Due to these changes, there is the possibility that the specificity and sensitivity of serological tests for COVID-19 diagnosis may be impacted (leading to false negatives) (31). In Figure 3, in the first red box, we show how we insert the custom epitope condition. In the second red box, we input an amino acid condition, N protein, 168–168 position range for a substitution for P (Proline) to S (Serine). By adding this condition, we are instructing the system to compute statistics only over the fraction of the selected population (all SARS-CoV-2, human host sequences in this example) that carry the specified amino acid change. In the Result section, green box (3), we observe that the mutated sequence ratios for the N4P5 epitope over the three defined populations are quite low (below 0.5%). Note that, if the ‘MUT FREQ’ is close to 1—when one of these three changes is present—no other change is carried within the epitope scope. Overall, the impacts on the epitope are minimal but attention should be paid, and further investigations are needed to proceed with its use for serologic assays.

### Example 2

Aiming to pave the way for designing novel vaccine candidates, Rakib *et al.* (32) propose to focus on epitopes located along the nucleocapsid (N) protein, especially for its high conservancy and dominant/long-lasting immune response [previously reported against SARS-CoV (33) and infectious bronchitis virus (34)]. From an immunological point of view, vaccine development has historically relied mostly on B cell immunity, but recent discoveries (35) revealed that T cell epitopes are also promising, leading to a more long-lasting immune response mediated by CD8+ T cells (thus recognizing viral peptides in the MHC class I area). By using EpiSurf in its Mode 2, we may perform a search on the updated corpus of IEDB deposited epitopes and then test their conservancy on selected populations. Drop-down menus may be employed to select epitopes on the ‘protein = N (nucleocapsid phosphoprotein)’, with ‘assay type = T cell’ and ‘hla restriction = HLA class I’. We then recommend selecting a high response frequency for positive assays (at least 0.2). Alternatively, one could select specific alleles from the ‘hla restriction’ menu and test epitopes only on a population of sequences from hosts that exhibit such alleles (in a statistically significant way).

**Figure 4.**
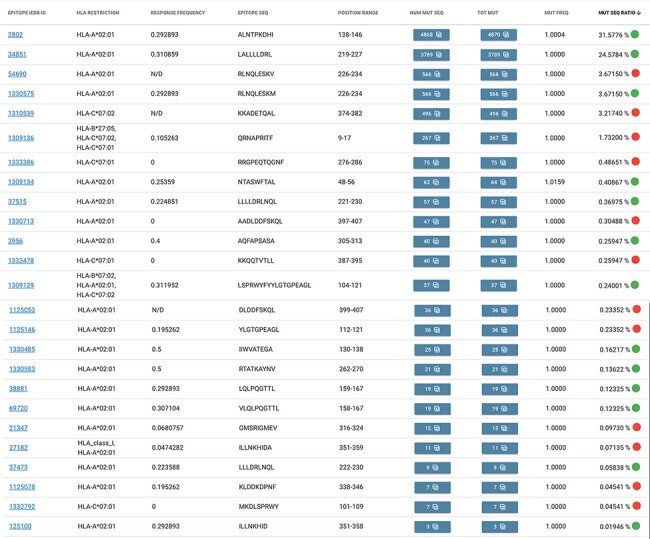
Result table of the query performed in Example 2: list of epitopes with their statistics descriptive of the mutation rate over the selected population (Campania, Italy). Color codes are used to discriminate between epitopes with a response frequency > 0.2 (green) or < 0.2 (red).

Suppose we are observing the Italian population of sequences from the Campania region (to date, most Italian sequences were sequenced here and GISAID depositions are about 15 000, as of 9 May 2021). From the Allele Frequency Net Database web portal (27), we gather that the most present (above a 40% threshold) alleles in the ‘Italy South Campania Region’ are DRB1*11 (49.6%), A*02 (43.0%) and C*07 (41.7%). In EpiSurf, we find no occurrence of T cell assay epitopes restricted to HLA-DBR1*11. However, we do find 20 epitopes restricted to HLA-A*02 and 7 epitopes restricted to HLA-C*07 (with two different subtypes). For observing the distribution of mutations over such epitopes we switch to Mode 3, which preserves our previous selections. By applying the epitope search, we obtain the table shown in Figure 4, where we appreciate the total of 25 epitopes (note that two of these, IEDB 1 309 129 and 1 309 136, match multiple HLA restriction filter conditions). Out of these, 13 of them exhibit a green mark, as their response frequency (for positive assays) is above the 0.2 threshold, whereas the other 12 show a red mark, meaning that users should carefully consider the statistics, as they may not be meaningful in the selected population. We also note that the most mutated epitope (ID 2802) has almost 4900 amino acid changes in the Campania population, most of which are of type L139F [also reported to modify protein flexibility and stability (30)].

### Example 3

In the last months of 2020, there has been interest in studying seven independent lineages circulating in the USA all having a change in the Glutamine (Q) amino acid at position 677 of the Spike protein. These all seemed to have originated and spread in the last few months (36). Q at 677 is more commonly mutated into Histidine (H)—leading to a non-radical change—however, in a considerable number of cases (predominantly in Texas), the change to Proline (P) has been observed, being this a radical change (Grantham distance = 76).

**Figure 5.**
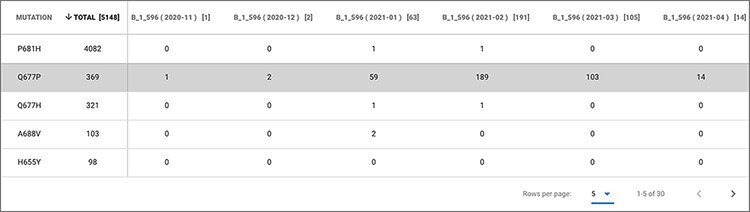
Epitope mutation statistics result of Example 3.

**Figure 6.**
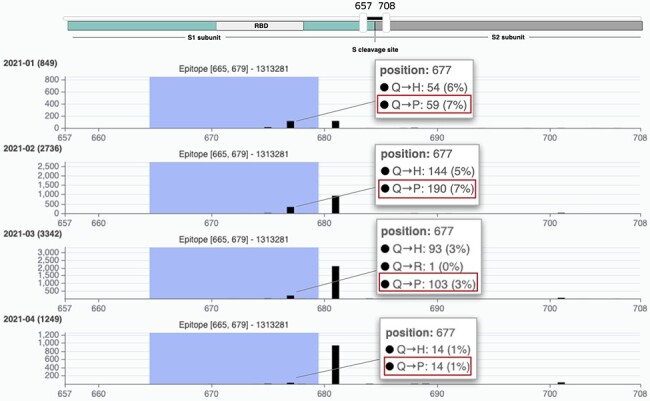
VirusViz compare functionality was applied to four groups of sequences collected in Texas in the first months of 2021 (see Example 3). We highlight a particular epitope that does not include the highly mutated 681 position (belonging to B.1.1.7 lineage, known as the UK variant of concern) but does include the position 677 and thus the Q766P mutation.

According to Hodcroft *et al.* (36), this change should be monitored as 677 is nearby to—although outside of—the furin binding pocket (polybasic site), important for the S1/S2 cleavage; therefore, hypothetically the presence of Proline in this precise spot could influence the cleavage of S1/S2. As this change is radical, with potentially interesting effects and geographically quite delimited, it serves as a good candidate to be monitored within EpiSurf. From our system, we choose the full sequence population of Texas (about 12 000 sequences, as of 9 May 2021), then—using Mode 3—we select epitopes located on the Spike protein that overlap the 677 position (obtaining 24 results) and finally, we set the condition of one substitution at position 677 into the Proline (P) alternative residue. As a result, we observe that all 24 epitopes exhibit 369 sequences where such change occurs. This set of sequences may be further inspected by clicking on any number in the *TOT MUT* column. The shown ‘Epitope mutation statistics’ functionality can be employed to group by lineage and collection month to observe—as shown in Figure 5—that all such sequences belong to lineage B.1.596 and that the Q677P change has been observed mostly between January and March 2021. A user may further investigate the selected Texas population by, for example, clicking on the ‘VirusViz All Epitopes’ button. The tool opens directly on the distribution of amino acid variants of the Spike protein. From the left menu ‘Highlight region’, users may select epitopes one at a time, thereby highlighting a specific position range in the bar plot. Note that immediately from the first visualization it is evident that 34% of the population exhibits the P681H amino acid change, feared for impacting the antibody recognition of linear SARS-CoV-2 epitopes, reducing Class 3 antibody recognition [even if this was only suggested in the non-peer reviewed literature (37) so far]. The user may then want to remove all epitopes that include this critical position. This may be achieved in the ‘Regions’ page, where epitopes are presented in the form of a list and can be dropped. Only two remain: IEDB ID 1 313 281 (position range 655–679) and 1 310 485 (position range 666–680). In the Population page, the user may observe that there has been a considerable number of depositions of sequences collected since 2021. It may be interesting to observe how the variant distributions behave (in terms of percentages) in the initial months of this year. By building different groups for the first 4 months of 2021, we produce a comparative visualization shown in Figure 6. Here we observe that the mutation Q677P decreases its occurrences in percentage, therefore diminishing the concerns on its possible effects on epitopes designed in this position range.

### Example 4

The focus of EpiSurf is on SARS-CoV-2; however, variation over epitopes of other viral species may be analyzed. Chen *et al.* (38) generated 12 monoclonal antibodies as experimental candidates to develop antibodies neutralizing Dengue Virus serotype 1 (DENV-1). They define an epitope on the domain III of the DENV-1 E protein, spanning from residues 346 and 360 with the sequence TQNGRLITANPIVTD, deemed as a highly conserved region among different genotypes of DENV-1.

The conservation of the epitope can be checked against EpiSurf sequences. We restrict our search to Dengue Virus 1 sequences deposited in the GenBank that were collected from human hosts before October 2017 (matching the publication date of the work from Chen *et al.*). We also select only complete sequences to ensure the accuracy of the variation calling algorithm, thus retrieving a total of 1665 sequences. By using Mode 1 of EpiSurf (Custom Epitopes), we then build the target epitope on the E protein with the range 346–360. As a result, we observe that 64 of the sequences exhibit at least one mutation (‘MUT FREQ’ of 1.06), representing the 3.84% of the total set (‘MUT SEQ RATIO’).

Chen *et al.* perform multiple sequence alignment of residues corresponding to the proposed epitope, thereby showing that it contains three conserved residues, namely G349, R350 and P356. By using the ‘Epitope mutation statistics’ panel, we can check if any mutation occurs at these specific positions; specifically, we find only one sequence with the G349D substitution and one with the P356H substitution. We can also confirm the presence of L351V, an additional amino acid substitution mentioned by Chen *et al.*, appearing in two sequences. Incidentally, we notice that the mutation T346I, not mentioned in their work, is the most present in the dataset (29 sequences) and could thus be further investigated.

## Discussion

 During 2020 and the beginning of 2021, fueled by the outbreak of the COVID-19 pandemics, huge interest has been focused on studying epitopes, parts of the SARS-CoV-2 sequence that can be recognized by vaccines, drugs and serological tests. For epitopes, IEDB is recognized as the most important, fully public repository, as of today collecting about 5000 epitopes of SARS-CoV-2 (along with many other viruses), well-described by means of attributes and search panels.

Several computational tools are used for supporting the prediction of epitopes. Our EpiSurf system covers a different need, as it provides a flexible interface for testing their conservancy, measured as the presence/absence of amino acid changes over epitope sequences. The unique aspect of EpiSurf is the ability to perform such conservancy testing by intersecting epitopes of interest, extracted by means of queries in the IEDB, against the amino acid changes that are present in arbitrarily selected viral sequences, e.g. by lineage, location or time of sequence collection. Such queries upon viral sequences can also be used against custom epitopes, freely entered by EpiSurf users.

Extensive analytical and visual support is offered to the users, including aggregations by metadata, statistics of mutated sequences and distribution plots (through our connection to VirusViz). EpiSurf aims to be a solid companion tool for researchers designing epitopes that need to rely on the big corpus of sequences available online.

## Data Availability

The code of EpiSurf is available on GitHub at https://github.com/DEIB-GECO/EpiSurf/ and on Zenodo at http://doi.org/10.5281/zenodo.5121287. The system is documented in the related WIKI at https://github.com/DEIB-GECO/EpiSurf/wiki/.
